# Clinical, Pathological, and Molecular Characteristics of CpG Island Methylator Phenotype in Colorectal Cancer: A Systematic Review and Meta-analysis

**DOI:** 10.1016/j.tranon.2018.07.008

**Published:** 2018-07-30

**Authors:** Shailesh M. Advani, Pragati Advani, Stacia M. DeSantis, Derek Brown, Helena M. VonVille, Michael Lam, Jonathan M. Loree, Amir Mehrvarz Sarshekeh, Jan Bressler, David S. Lopez, Carrie R. Daniel, Michael D. Swartz, Scott Kopetz

**Affiliations:** * Division of Gastrointestinal Medical Oncology, The University of Texas MD Anderson Cancer Center, Houston, TX 77030; † Human Genetics Center, Department of Epidemiology, Human Genetics, and Environmental Sciences, School of Public Health, The University of Texas Health Science Center at Houston, Houston, TX 77030; ‡ Lombardi Cancer Center, Division of Oncology, Georgetown University School of Medicine, Washington, DC 20007; § Radiation Epidemiology Branch, Division of Cancer Epidemiology and Genetics, National Cancer Institute, Rockville, MD 20850; ¶ Department of Biostatistics and Data Science, School of Public Health, The University of Texas Health Science Center at Houston, Houston, TX 77030; # Library, School of Public Health, The University of Texas Health Science Center at Houston, Houston, TX 77030; ** Division of Medical Oncology, BC Cancer, Vancouver, BC, Canada, V5Z 4E6; †† Division of Urology, UTHealth McGovern Medical School, Houston, TX 77030; ‡‡ Department of Epidemiology, Division of Cancer Prevention and Population Sciences, The University of Texas MD Anderson Cancer Center, Houston, TX 77030

## Abstract

*BACKGROUND:* CpG island methylator phenotype (CIMP) tumors, comprising 20% of colorectal cancers, are associated with female sex, age, right-sided location, and BRAF mutations. However, other factors potentially associated with CIMP have not been robustly examined. This meta-analysis provides a comprehensive assessment of the clinical, pathologic, and molecular characteristics that define CIMP tumors. *METHODS:* We conducted a comprehensive search of the literature from January 1999 through April 2018 and identified 122 articles, on which comprehensive data abstraction was performed on the clinical, pathologic, molecular, and mutational characteristics of CIMP subgroups, classified based on the extent of DNA methylation of tumor suppressor genes assessed using a variety of laboratory methods. Associations of CIMP with outcome parameters were estimated using pooled odds ratio or standardized mean differences using random-effects model. *RESULTS:* We confirmed prior associations including female sex, older age, right-sided tumor location, poor differentiation, and microsatellite instability. In addition to the recognized association with BRAF mutations, CIMP was also associated with PIK3CA mutations and lack of mutations in KRAS and TP53. Evidence of an activated immune response was seen with high rates of tumor-infiltrating lymphocytes (but not peritumoral lymphocytes), Crohn-like infiltrates, and infiltration with *Fusobacterium nucleatum* bacteria. Additionally, CIMP tumors were associated with advance T-stage and presence of perineural and lymphovascular invasion. *CONCLUSION:* The meta-analysis highlights key features distinguishing CIMP in colorectal cancer, including molecular characteristics of an active immune response. Improved understanding of this unique molecular subtype of colorectal cancer may provide insights into prevention and treatment.

## Introduction

Colorectal cancer (CRC) is a major public health problem as more than 1 million new cases are diagnosed worldwide every year [1]. CRCs represent a heterogeneous group of tumors characterized by complex multifactorial phenotypes that are influenced by host factors [2]. These include diet, environmental, microbial, genetic, and epigenetic factors, as well as metabolic and other exposures [3]. In addition, genomic instability is an important molecular event in the development of CRC, encompassing chromosomal instability, microsatellite instability (MSI), and aberrant DNA methylation [4]. Two main pathways have been characterized in the development of CRC. The suppressor pathway is involved in 80% of CRC cases and is characterized by chromosomal instability. The remaining 15%-20% of CRCs likely arise from the serrated adenoma pathway, which is often associated with epigenetic silencing of the mismatch repair gene *MLH1*. Aberrant DNA methylation is a hallmark of human cancer and consists of both global DNA hypomethylation and site-specific DNA hypermethylation [5], [6]. CpG island methylator phenotype (CIMP), thought to be a precursor to the serrated adenoma pathway, represents a subset of CRCs characterized by significant hypermethylation of CpG islands of tumor suppressor genes, leading to their inactivation and thereby promoting tumor progression [6], [7]. CIMP is a distinct phenotype characterized by high promoter methylation of several genes including MINT clones, p16, THBS, and MLH1 as first shown by Toyata et al. in CRC tissues and is characterized by key clinical, pathologic, and molecular characteristics, including female sex, old age, high MSI, *BRAF* mutations, and right-sided tumor location [7], [8].

Since the discovery of CIMP in 1999, various tools and methodologies have been developed to quantify methylation and CIMP status in CRC tumors. The first panel used MINT or specific methylated in tumor markers to study gene-specific methylation [7]. Subsequently, the Weissenberg panel and Ogino panel were developed and are widely used to study CIMP among CRC patients [9]. In 2016, a systematic review of the clinical, pathologic, and molecular characteristics of CIMP tumors showed that CIMP was associated with *BRAF* mutations, high MSI, female sex, right-sided tumor location, and age [8]. The researchers classified CIMP identification methodologies into four groups: Classical panel, Weisenberg panel, Combination panels, and Human Methylation Arrays. Using these classifications, the researchers found that the association of CIMP with the various clinical, pathologic, and molecular characteristics differed in magnitude and direction of association based on methodological classification [8]. However, their study restricted their inclusion to these methodological subtypes.

With advancement in technology and better understanding of epigenetic basis of disease, classification of CIMP varies based on definition and methodology, and is generally classified as CIMP-High (CIMP-H), CIMP-Low (CIMP-L) and CIMP-0, or CIMP-Positive and CIMP-Negative [10]. However previous studies have also reported no differences in CIMP-L and CIMP-0, and classified them as non-CIMP or CIMP-0 [9], [11]. With the growing influence of translational research and molecular pathology, integrating molecular, lifestyle, and demographic characteristics is key to understanding the carcinogenic pathways that underlie different subtypes of cancer. This systematic review aims to provide an update to the previous literature with a comprehensive assessment of the clinical, pathologic, and molecular characteristics of CIMP tumors in CRC. In addition, we have performed a meta-analysis of all factors with adequate available data to clarify the direction of the associations. Our results will aid in defining the key characteristics of CIMP tumors and ultimately provide the clinical community with necessary information to identify patients with CIMP tumors. Improving understanding of these associations can identify potential pathways that characterize CIMP and help researchers develop pathway-specific cancer prevention and treatment strategies.

## Methods

This systematic review follows the publishing guidelines as set forth by the Preferred Reporting Items for Systematic Reviews and Meta-Analyses (PRISMA) [12]. It has been registered with PROSPERO (registration number CRD42016034181). Eligibility criteria were determined a priori and required that studies examined the association of CIMP with clinical, pathologic, and molecular characteristics among patients with sporadic CRC. We excluded all studies that focused on hereditary CRC syndrome (familial adenomatous polyposis or lynch syndrome), studies focusing on other cancers or premalignant CRC lesions such as adenomas or polyps, and articles that did not have a clear description of the measurement or quantification of CIMP. Only original research articles published in English-language were included; comments, editorials, dissertations, conference proceedings, reviews, etc., were excluded. The three main concepts that made up our search were CIMP, sporadic CRC, and clinical/pathologic and molecular characteristics.

### Search Strategy

We searched Medline (Ovid), PubMed (NLM), Embase (Ovid), and PsycINFO (Ovid) with the help of a health sciences librarian (H.V.) with systematic review experience who developed all searches. The initial searches were completed in April 2016; an updated PubMed search was completed April 5, 2018. A combination of MeSH terms and title, abstract, and keywords was used to develop the initial Medline search. The search was then adapted to search other databases. Supplemental Material (Appendix 1) provides the search strategies used for each database. RefWorks (ProQuest) was used to store all citations found in the search and to check for duplicates. Search strategies and results were tracked using one of a series of Microsoft Excel workbooks designed specifically for systematic reviews by the health sciences librarian (H.V.) [13].

An online random-number generator (https://www.random.org/integers/) was used to create a random sample of 146 numbers that were then input into an Excel workbook designed specifically for the interrater reliability test [13]. These numbers corresponded to line numbers within the Excel workbook which resulted in a random sample of titles and abstracts; authors and journal titles were not included in the sample. Two authors (S.A., P.A.) independently screened the sample and reached moderate agreement (Cohen's *κ* = 0.77) [14]. Screening discrepancies of the sample dataset were resolved at which time they then independently screened all titles and abstracts, still blinded to authors and journal titles, using an Excel workbook designed specifically for this step of the systematic review process. Data were compiled into a single Excel workbook, and consensus was reached on items in which there was disagreement. Articles considered for inclusion were independently reviewed by the two authors (S.A., P.A.), and consensus was reached by discussion on any disagreements for inclusion.

### Data Abstraction

The primary author (S.A.) extracted the following data for each study: basic study characteristics, including study design, primary author of the study, cohort description, and country of study; panel markers and/or methodology used to measure CIMP, cutoff for classifying various CIMP groups, and prevalence of each CIMP subgroup; patient demographics including age and gender; clinical, pathologic, and molecular characteristics; and the prevalence of each characteristic across CIMP subgroups. Most common classification included classifying CIMP into two groups: CIMP-H and CIMP-0. However certain studies classified CIMP as CIMP-H, CIMP-L, and CIMP-0. Few studies also classified CIMP as CIMP-Positive (CIMP+) and CIMP-Negative (CIMP−). For consistency, we have labeled CIMP-H and CIMP-positive as CIMP-H, and CIMP-L, CIMP-0, and CIMP-negative as CIMP-0.

### Meta-Analysis

Pooled odds ratios (ORs) and 95% confidence intervals (CIs) were calculated for the association of CIMP with the various characteristics. Additionally, for continuous variables (age), a standardized mean difference was measured for CIMP groups. A random-effects model was utilized to measure these associations. A *P* value of less than .05 was considered statistically significant. A measure of heterogeneity (*I*^2^) was also calculated. In addition, the Egger test was used to measure bias due to small size effects. Funnel plots were generated to study the distribution of effect sizes. In addition, the pooled prevalence of each characteristic was measured in the CIMP-H and CIMP-0 subgroups wherever possible.

#### Quality Assessment

We performed quality assessment on included studies. For cohort and case control studies, the Newcastle-Ottawa Scale was utilized [15]. The scale assesses quality of included studies on three groups: Selection; Comparability, and Assessment. For cohort studies, these include selection of cohort, comparability of exposed and nonexposed cohort, and assessment of outcome and follow-up data. For case control, these include selection of cases and controls, comparability of cases and controls, and ascertainment of exposure, including ascertainment of cases and controls and response rates. Reviewers rate studies on scale of 0-4 for selection, scale of 0-2 for comparability, and scale of 0-3 for ascertainment respectively.

## Results

**Figure 1:**
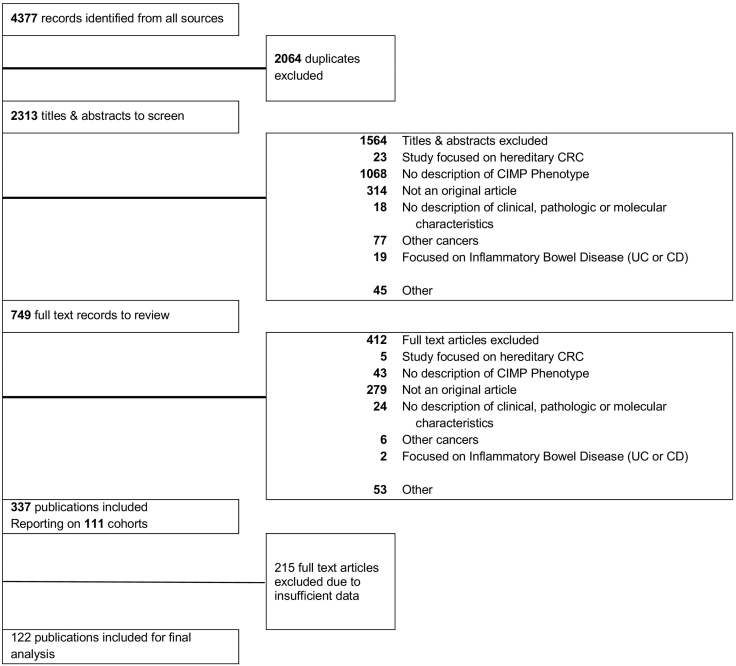
Preferred Reporting Items for Systematic Reviews and Meta-Analyses flowchart outlining the literature search process. *CRC*, colorectal cancer; *CIMP*, CpG island methylator phenotype.

Our search identified 4377 abstracts for initial screening. After removal of duplicates, a total of 2313 abstracts were screened by two authors (S.A., P.A.). The Cohen *κ* statistic was 0.77, indicating good agreement between the two authors. We identified 749 abstracts in this process eligible for full-text review. After full-text review of 749 studies, 337 publications were selected for final data abstraction process. The main reasons for exclusion included conference abstracts or studies focused on other cancers or other CRC lesions such as premalignancies. Figure 1 outlines the entire screening process and reasons for exclusion. Of the 337 publications; we included 122 for final meta-analysis in this project.

### Study Characteristics

We identified 122 publications; 113 publications utilized data from cohort studies, 8 utilized case control studies, and 1 utilized data from a randomized clinical trial. Most studies were from the United States, Japan, and Australia. For each characteristic, the data distribution across CIMP groups was assessed from the cohort with largest sample size identified in the literature to minimize bias due to repetitive data abstraction from the same patient population.

**Figure 2:**
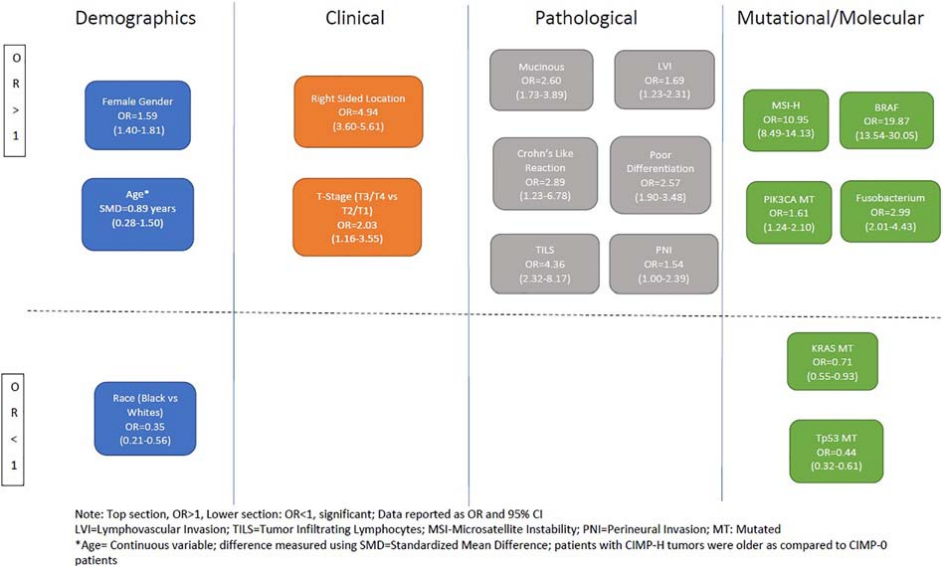
Significant associations of CIMP with demographic, clinical, genetic and molecular, or pathologic characteristics as identified in the meta-analysis. *OR*, odds ratio; *SMD*, standardized mean difference.

We obtained data for multiple characteristics with its association across CIMP groups. However, to make meaningful conclusions, we performed meta-analysis on 27 characteristics described below. Of 337 articles, 215 had insufficient data for inclusion into meta-analysis for factors assessed in this project, leaving 122 included publications for inclusion in final analysis. Below is a summary of the demographic, clinical, pathologic, mutational, and molecular characteristics that were reviewed and studied for their association with CIMP in the literature. Table 1 provides a summary of our meta-analysis including pooled OR and summary pooled estimates (prevalence) of various characteristics across CIMP-H group. Figure 2 provides a summary of significant associations identified in our analysis. Appendix 2 provides forest plots for each of the characteristics and funnel plots for assessment of bias.

#### Demographic Characteristics

We explored the association of CIMP with demographic characteristics including age at diagnosis, race/ethnicity, gender, and family history of CRC. The association of CIMP with age at diagnosis (continuous variable) was explored in 16 studies representing a total of 3948 cases [16], [17], [18], [19], [20], [21], [22], [23], [24], [25], [26], [27], [28], [29], [30], [31]. While CIMP-H patients were older at diagnosis compare to CIMP-0 patients (*P* < .001), the magnitude of this difference was modest (standardized mean difference = 0.89 years, 95% CI = 0.28-1.50 years), confirming previous association of CIMP-H with older age. We also studied the association of CIMP with race/ethnicity across four studies [32], [33], [34], [35]. Using non-Hispanic whites as a reference group, we identified that patients of black race had substantially lower likelihood of having a CIMP-H tumor (pooled OR = 0.35, 95% CI = 0.21-0.56). In contrast, the likelihood of CIMP in Hispanic patients was indistinguishable from non-Hispanic white patients (OR = 1.04, 95% CI = 0.70-1.55). The association of CIMP-H with gender was studied across 56 studies, representing a total of 22,950 CRC patients [9], [16], [17], [19], [20], [21], [22], [24], [25], [27], [28], [30], [31], [33], [36], [37], [38], [39], [40], [41], [42], [43], [44], [45], [46], [47], [48], [49], [50], [51], [52], [53], [54], [55], [56], [57], [58], [59], [60], [61], [62], [63], [64], [65], [66], [67], [68], [69], [70], [71], [72], [73], [74], [75], [76], [77]. The pooled prevalence of CIMP-H in males and females was 18% (95% CI = 15%-20%) and 26% (95% CI = 22%-29%), respectively, indicating a predisposition for CIMP-H tumors towards female gender (pooled OR = 1.59, 95% CI = 1.40-1.81)***.*** Finally, we did not observe any association of CIMP with family history of cancer across four studies [35], [47], [78], [79]. The pooled OR for family history of CRC in the CIMP-H subgroup compared with the CIMP-0 subgroup was 0.93 (95% CI = 0.72-1.20, *I*^2^ = 29.6%). Hence, our analysis identified a strong inverse association between CIMP-H and black race and confirmed previously reported associations of CIMP with older age and female sex. See Appendix 2, Figures 1-10, for forest and funnel plots for association of CIMP with demographic characteristics.

#### Clinical Characteristics

We studied the association of CIMP with tumor location, T staging, N staging, M staging, overall stage, well as synchronous CRC and presence of liver metastases. The association of CIMP with tumor location (right colon vs left colon + rectum) was investigated in 56 studies, representing a total of 20,782 CRC patients [16], [17], [19], [23], [24], [25], [28], [29], [30], [31], [33], [36], [37], [38], [39], [40], [41], [42], [43], [44], [45], [47], [48], [50], [51], [54], [55], [56], [57], [58], [59], [60], [61], [62], [64], [65], [66], [67], [68], [69], [70], [72], [73], [74], [76], [77], [78], [80], [81], [82], [83], [84], [85], [86], [87], [88]. The pooled OR for right-sided location (as opposed to left-sided or rectal location) in the CIMP-H subgroup compared with the CIMP-0 subgroup was 4.84 (95% CI = 3.60-5.61, *I*^2^ = 80%). The association of CIMP with T staging was investigated across 11 studies, representing 3255 cases [20], [21], [36], [50], [51], [62], [65], [69], [71], [77], [89]. The pooled OR for T-stage (T3/T4) (as compared to T1/T2) in the CIMP-H subgroup compared with CIMP-0 subgroup was 2.03 (95% CI = 1.16-3.55, *I*^2^ = 61%). The association of CIMP with overall stage was investigated across 35 studies, representing a total of 12,668 CRC patients [16], [17], [20], [22], [24], [28], [33], [37], [38], [40], [41], [44], [45], [47], [48], [53], [54], [55], [56], [57], [58], [59], [63], [65], [66], [68], [72], [73], [74], [78], [89], [90], [91], [92], [93]. The pooled OR for stage III/IV disease (compared to I/II disease) in the CIMP-H subgroup compared with the CIMP-0 subgroup was 1.01 (95% CI = 0.87-1.17, *I*^2^ = 48.1%). In pooled analysis, no association was observed for CIMP with N staging [OR for N1/2 vs N0 = 1.14 (95% CI = 0.87-1.49, *I*^2^ = 38.9%)] [16], [20], [21], [25], [36], [39], [50], [51], [61], [65], [69], [71], [72], [75], [77], [83], [94] and M staging [OR for M1/M2 vs M0 = 0.92 (95% CI = 0.56-1.57, *I*^2^ = 38.5%)] [20], [39], [45], [53], [65], [95]. Similarly, no association of CIMP was observed with synchronous CRC [58], [96], [97] or presence of liver metastases [61], [98], [99].

**Table 1:** Summary Estimates for Association of CIMP Status with Clinical, Pathological, Molecular, and Mutational Characteristics Using Meta-Analysis Approach

Characteristic	No. of Studies	No. of CRC cases	Pooled OR/SMD	95% CI	*I*^2^, %	Egger *P*	Prevalence, % of CIMP-H	95% CI for prevalence, %
Demographic								
Age at diagnosis	16	3948	0.89 years	0.28-1.50	97.9	.35	NA	NA
Gender	56	22,950						
Male			Ref				18	15-20
Female			1.59	1.40-1.81	58.1	.36	26	22-29
Race								
NHW	4	7948	Ref				93	92-95
NHB			0.35	0.21-0.56	0	.7	2	0-5
Hispanics			1.04	0.70-1.55	0	.7	3	1-5
Family history of CRC	4	4291						
No			Ref				46	33-58
Yes			0.93	0.72-1.20	29.6	.23	13	10-16
Clinical								
T staging								
T1/2	11	3255	Ref				8	4-11
T3/4			2.03	1.16-3.55	60.8	.48	18	13-23
N staging								
N0	17	3502	Ref				16	12-20
N1/2			1.14	0.87-1.49	38.9	.05	17	13-21
M staging								
M0	6	1343	Ref				23	12-34
M1			0.92	0.65-1.57	38.5	.10	18	10-26
Overall stage								
I-II	35	12,668	Ref				Stage I=13	11-16
III-IV			1.01	0.87-1.17	48.1	.83	Stage II=19	16-22
							Stage III=18	16-21
							Stage IV=16	12-19
Localization								
Left	56	20,782	Ref				11	10-13
Right			4.94	3.60-5.61	80.0	.06	36	32-41
Synchronous CRC								
No	3	1243	Ref				16	13-19
Yes			1.66	0.66-4.08	45.5	.5	22	8-37
Liver mets								
No	3	395	Ref				29	17-40
Yes			0.91	0.52-1.60	23.8	.02	26	11-41

Pathological Characteristics								
LVI								
No	9	1572	Ref				23	15-31
Yes			1.69	1.23-2.31	18.1	.75	32	22-43
TILS								
No	5	2053	Ref				14	8-19
Yes			4.36	2.32-8.17	79.9	.39	42	21-63
PLS								
No	5	2270	Ref				12	9-16
Yes			1.74	0.92-3.30	75.8	.32	22	12-32
Vascular invasion								
No	9	2067	Ref				15	9-21
Yes			1.20	0.77-1.88	35.7	.47	11	8-15
PNI								
No	4	890	Ref				35	18-52
Yes			1.54	1.00-2.39	0	.20	39	28-51
Crohn-like infiltrate								
No	4	2398	Ref				11	7-15
Yes			2.89	1.23-6.78	88.4	.68	28	9-48
Signet ring cell histology								
No	4	1673	Ref				14	6-22
Yes			3.10	0.98-9.86	46.5	.44	44	36-53
Mucinous histology								
No	21	6297	Ref				15	12-19
Yes			2.60	1.73-3.89	80.9	.86	30	24-37
Differentiation								
Well/moderate			Ref				20	16-24
Poor	26	7095	2.57	1.90-3.48	70.4	.59	37	30-45

Mutational and Molecular Characteristics								
Tp53 mutation								
WT			Ref				30	23-38
MT	19	7021	0.44	0.32-0.61	78.7	.04	14	11-17
PIK3CA mutation								
WT	6	3285	Ref				16	13-19
MT			1.61	1.24-2.10	12.5	.49	22	18-26
APC mutation								
WT	5	1641	Ref				38	18-58
MT			0.56	0.16-1.84	93.3	.81	26	9-42
MSI								
MSS	54	21,994	Ref				15	13-16
MSI-H			10.95	8.49-14.13	83	.21	61	55-66
BRAF mutation								
WT	44	20,615	Ref				12	10-13
MT			20.17	13.54-30.05	88.4	.03	68	57-79
KRAS mutation								
WT	41	16,784	Ref				22	19-25
MT			0.71	0.55-0.93	81.3	.48	17	14-20
*Fusobacterium nucleatum*								
Negative	4	1392	Ref				17	10-24
Positive			2.99	2.01-4.43	0	.65	38	17-59

*NHW*, non-Hispanic whites; *NHB*, non-Hispanic blacks; *WT*, wild type; *MT*, mutated; *MSS*, microsatellite stable; MSI, microsatellite-instable high; *CRC*, colorectal cancer.

Our results confirm the previously reported association of CIMP with right-sided tumor location. Additionally, we identified CIMP-H tumors to be associated with advanced T-stage (T3/T4). In summary, our results identified no association between CIMP and overall stage, as well as N or M staging, synchronous CRC, or the presence of liver metastases. See Appendix 2, Figures 11-24, for funnel plots and forest plots for association of clinical factors with CIMP.

#### Pathologic Characteristics

We studied the association of CIMP with key pathological characteristics in CRC tumors including lymphovascular invasion (LVI), tumor-infiltrating lymphocytes (TILS), peritumoral lymphocytes (PLS), vascular invasion, perineural invasion (PNI), presence of Crohn-like infiltrates, mucinous histology, and signet ring cell histologic characteristics. We investigated the association of CIMP with LVI in nine studies, representing 1572 CRC cases, and identified CIMP-H tumors to be associated with LVI as compared to CIMP-0 tumors (OR = 1.69, 95% CI = 1.23-2.31, *I*^2^ = 18.1%) [30], [38], [43], [45], [50], [59], [62], [65], [72]. The association of CIMP with TILS was investigated in five studies, representing 2015 CRC cases [19], [38], [69], [100], [101]. The pooled OR for presence of TILS in the CIMP-H subgroup compared with the CIMP-0 subgroup was 4.36 (95% CI = 2.32-8.17, *I*^2^ = 79.9%). A borderline association was identified for CIMP-H tumors with PLS [OR = 1.74 (95% CI = 0.92-3.30, *I*^2^ = 75.8%)] in pooled analysis of five studies [44], [69], [77], [100], [101] and with signet ring histology [OR = 3.10 (95% CI = 0.98-9.86, *I*^2^ = 46.5%)] across four studies [52], [58], [102], [103]. We found borderline association of CIMP with PNI across four studies [30], [38], [50], [62]. The pooled OR for PNI across CIMP-H groups was 1.54 (95% CI = 1.00-2.39, *I*^2^ = 0%). CIMP-H tumors were also found to be associated with presence of Crohn-like infiltrate [OR = 2.89 (95% CI = 1.23-6.78, *I*^2^ = 88.4%] in pooled analysis of four studies [44], [69], [100], [101]. No associations were observed for CIMP status with vascular invasion [OR = 1.20 (95% CI = 0.77-1.88, *I*^2^ = 35.7%)] across nine studies [45], [52], [65], [69], [71], [72], [77], [83], [104]. Finally, we also confirmed association of CIMP-H tumors with mucinous histology [OR = 2.60 (95% CI = 1.73-3.89, *I*^2^ = 80.90%)] in pooled analysis of 21 studies [19], [25], [30], [36], [39], [41], [43], [44], [54], [56], [58], [65], [66], [72], [76], [77], [81], [83], [84], [103], [105] and with poor differentiation [OR = 2.57 (95% CI = 1.90-3.84, *I*^2^ = 70.4%)] in pooled analysis of 26 studies [17], [25], [30], [33], [36], [37], [39], [41], [43], [44], [45], [50], [52], [53], [54], [58], [67], [69], [72], [74], [75], [76], [81], [84], [92], [105]. In summary, our results indicated that CIMP was associated with TILS, Crohn-like infiltrates, and LVI. CIMP-H was common in patients with signet ring cell histologic characteristics, but the association was not significant. See Figures 25-42 for funnel plots and forest plots for association of pathological factors with CIMP in Appendix 2.

#### Mutational and Molecular Characteristics

We studied the association of CIMP with key mutations in CRC pathogenesis including mutations in *KRAS*, *BRAF*, *PIK3CA*, *TP53*, and *APC* as well as microsatellite instability (MSI) and presence of *Fusobacterium nucleatum*. We calculated both pooled prevalence and ORs for each of these associations as shown in Table 1. Using meta-analysis, the pooled prevalence of CIMP-H in *BRAF* MT, *BRAF* WT, *KRAS* MT, *KRAS* WT, *PIK3CA* MT, *PIK3CA* WT, *TP53* MT, *TP53* WT, *APC* MT, and *APC* WT groups was 68%, 12%, 17%, 22%, 22%, 16%, 14%, 30%, 26%, and 38%, respectively. Similarly, the pooled prevalence of CIMP-H in MSI-H and MSS groups was 61% and 15%, respectively. The prevalence of high levels of *Fusobacterium nucleatum* in CIMP-H tumors was 38%, and the prevalence of low levels of FB in CIMP-H tumors was 15%.

CIMP-H was found to be associated with *BRAF* Mutation [OR = 20.17 (95% CI = 13.54-30.05, *I*^2^ = 83%)] [16], [17], [19], [22], [24], [25], [26], [28], [33], [37], [39], [40], [41], [42], [47], [48], [49], [52], [58], [59], [60], [62], [64], [66], [68], [69], [70], [72], [77], [78], [82], [83], [84], [93], [95], [106], [107], [108], [109], [110], [111], [112], [113], [114] and PIK3CA mutation [OR = 1.61 (95% CI = 1.24-2.10, *I*^2^ = 12.5%)] [55], [76], [115], [116], [117], [118]. An inverse association of CIMP-H tumors was observed with TP53 mutation [OR = 0.44 (95% CI = 0.32-0.61, *I*^2^ = 78.7%)] [24], [41], [44], [53], [55], [62], [64], [69], [74], [78], [83], [100], [105], [113], [119], [120], [121], [122], [123] and KRAS mutation [OR = 0.71 (95% CI = 0.55-0.93, *I*^2^ = 81%)] [16], [19], [21], [23], [24], [25], [26], [31], [33], [37], [38], [39], [40], [43], [47], [48], [52], [53], [55], [59], [60], [62], [67], [69], [70], [71], [72], [74], [76], [77], [78], [83], [84], [105], [107], [110], [111], [113], [121], [124], [125]. No associations were observed with APC mutation [OR = 0.56 (95% CI = 0.16-1.84, *I*^2^ = 93.3%)] [32], [55], [78], [113], [126]. We also confirmed previously reported association of CIMP-H with microsatellite instability (MSI-H) status [OR = 10.95 (95% CI = 8.49-14.13, *I*^2^ = 83%)] among CRC patients [16], [17], [19], [20], [23], [25], [28], [33], [34], [36], [38], [39], [40], [41], [43], [44], [47], [48], [50], [55], [58], [59], [60], [64], [65], [66], [67], [68], [70], [72], [74], [77], [80], [81], [82], [84], [95], [107], [109], [110], [111], [112], [127], [128], [129], [130], [131], [132], [133], [134], [135], [136], [137], [138]. Finally, we also observed a positive association of CIMP-H tumors with presence of high levels of fusobacterium nucleatum [OR = 2.99 (95% CI = 2.01-4.43, *I*^2^ = 0%)] [3], [64], [139], [140].

In summary, our results showed that CIMP was associated with *PIK3CA* mutations and inversely associated with *TP53* mutations. CIMP was also associated with *Fusobacterium nucleatum*. In addition, we validated the previously reported association of CIMP with *BRAF* mutations, MSI, and *KRAS* mutations. No association was observed between CIMP and *APC* mutations. See Figures 43-56 for funnel plots and forest plots for association of mutational/molecular factors with CIMP in Appendix 2.

### Quality Assessment

A visual assessment of funnel plots identified possible evidence of publication bias in association of CIMP with BRAF mutation, Tp53 mutation, gender, differentiation, and location. Egger's regression test was utilized to study whether studies with small sample size introduced possible publication bias in our analysis. We found evidence of possible publication bias due to low sample size in the association of CIMP with N staging (*P* = .05), liver metastases (*P* = .02), BRAF mutation (*P* = .04), and *TP53* mutation (*P* = .04). However, for other factors, possible different factors could play a role in introducing heterogeneity (*I*^2^ values of >50%) or publication bias, including selective reporting (of outcomes or exposures), differences in CIMP methodologies across studies, sampling variation, or by chance alone. To assess quality of studies included, refer to Appendix 3 (Cohort Studies) and Appendix 4 (Case Control Study) for quality assessment of included studies using the Newcastle-Ottawa scale.

## Discussion

Our systematic review confirmed the previously reported association of CIMP with high MSI, *BRAF* mutations, lack of *KRAS* mutations, poor differentiation, mucinous histologic characteristics, right-sided tumor location, female sex, and older age, In addition to this, to the best of our knowledge, we are the first to confirm association of CIMP with T staging (T3/T4), TILS, Crohn-like infiltrates, LVI, wild-type *TP53*, *PIK3CA* mutations, and high levels of *Fusobacterium nucleatum*, and an inverse association with black race using meta-analysis. In addition, CIMP was common among patients with signet ring cell histologic characteristics and those with PNI. CIMP in CRC provides a unique opportunity to study molecular mechanisms that lead to epigenetic changes in cancer and the contributions of these changes to the development of the disease [141].

Consistent with previous reports, we found that CIMP was associated with high MSI and *BRAF* mutations. These are thought to arise as events in the serrated adenoma pathway. *BRAF* mutations are thought to be early events in CIMP cancers, inhibiting normal apoptosis of colonic epithelial cells [11]. CIMP also seems to be more likely to develop when a *BRAF* mutation is present and the polyp is in the proximal colonic environment [142]. Other studies have hypothesized that the pathways of development differ between high-MSI cancers with *BRAF* mutations and microsatellite-stable cancers with *BRAF* mutations [143]. High-MSI CRCs may develop from a subset of hyperplastic polyps (which often have *BRAF* mutations and CIMP-H), and microsatellite-stable cancers with *BRAF* mutations may develop from adenomas with *BRAF* mutations. Alternatively, both types may share a similar initial pathway but diverge with respect to clinical aggressiveness; methylation of hMLH1 may occur in a subset of tumors that then develop high MSI [143]. In experimental models, CIMP-dependent DNA hypermethylation and transcriptional inactivation of IGFBP7 were shown to mediate *BRAF* V600E-induced cellular senescence and apoptosis [141]. Future prospective studies should explore this relationship in patients undergoing screening colonoscopy to determine the sequence of events.

We found that CIMP was associated with *PIK3CA* mutations. *PIK3CA* mutations occur in 10%-30% of CRC patients [144]. A mutation in *PIK3CA* stimulates the AKT (protein kinase B) pathway and promotes cell growth in various cancers, possibly through increased expression of fatty acid synthetase (FASN) [145]. FASN is an important regulator of energy balance and has been shown to be associated with cancer development [146]. A previous study by Nosho et al. identified higher levels of FASN expression in CIMP-H versus non-CIMP tumors; however, this should be further explored as a possible mechanism underlying the association of CIMP with *PIK3CA* [146].

Immunotherapy, as a tool for cancer management, has gained significant importance. Additionally, activation of immune system and subsequent immune reaction play an important role in tumor microenvironment in suppressing tumor development and progression [147]. The presence of TILS provides evidence that the host's immune system is attempting to eliminate the tumor, and this is an important favorable prognostic factor in CRC [148], [149]. Specific subsets of TILS (CD57^+^, CD8^+^, CD45RO^+^, or FOXP3^+^ cells) have been associated with improved clinical outcome in CRC [150]. TILS can trigger preferential lysis of cancer cells by recognizing enhanced expression of abnormally expressed antigens presented in the context of HLA molecules [151]. Recent molecular classification by TCGA identifies CIMP− tumors to be associated with CMS 1 phenotype which involves CRC tumors with strong immunogenic response to tumors and favorable survival [97]. In recent years, immunotherapy as a tool to stimulate immune system to target cancer and work to change tumor microenvironment has rapidly gained significant importance, and MSI-H cancers are favorable targets for immunotherapy-related clinical trials including pembrolizumab [152], [153]. Previous studies have also reported MSI cancers to be associated with expression of programmed cell death (PD1) intraepithelial lymphocytes and have shown favorable response on anti-PD1 therapy [154], [155]. The presence of TILS is considered a hallmark of MSI-high cancers, possibly due to truncated peptides produced by frameshift mutations in MSI-high cancers that have been shown to be immunogenic and to contribute to the host immune response and improved survival [156]. Although no association has been identified between CIMP and specific levels of TILS like FOXP3 levels or CD8 levels, this association of CIMP with TILS highlights possible role of the immune system in CIMP CRC and should be further explored by MSI status [156]. This also makes CIMP+ tumors potential targets for immunotherapy trials including anti-PD1 therapy as CIMP-H has been shown to be associated with PD-1–positive T cells in subset of MSI-H cancers [157]. Recently, the TIME (Tumor Immunity in the MicroEnvironment) classification system was developed based on high/low levels of tumor CD274 (PD-L1) expression and presence/absence of TILS to classify cancer subtypes [158], [159]. Hanada et al. identified CIMP-H tumors to be associated with TIME-2 and 3 subtypes characterized by presence of TILS [147]. Similarly, presence of Crohn-like infiltrate indicates association of CIMP with peritumoral lymphoid aggregates and role of immune system in MSI and CIMP cancers [160]. Crohn-like infiltrates confer favorable prognosis in CRC, indicating that the host immune response plays a role in preventing cancer progression and a possible mechanism affecting prognosis in CIMP tumors.

Microbiome has shown to play a distinct role in cancer development and progression, including response to chemotherapeutic agents [161], [162]. Our analysis identified that CIMP was associated with high levels of *Fusobacterium nucleatum*. *Fusobacterium* species are highly heterogeneous and opportunistic pathogens and have been linked to periodontitis, appendicitis, and inflammatory bowel diseases [64]. Clostridium *Fusobacterium nucleatum* is an anaerobic commensal, thought to provide a microenvironment for survival of CRC cells in the gut, especially in CIMP-H CRC [64], [163]. Preliminary studies have also identified *Fusobacterium nucleatum* to be involved in development of CRC through the serrated adenoma pathway [164]. In pilot studies, evidence suggest that fusobacterium may play a key role in CRC development and modulate response to chemotherapy among CRC patients; however, these results have limited generalizability due to their small sample size and need further validation in future prospective studies [165], [166], [167]. *Fusobacterium* species have been shown to have particular characteristics, including invasiveness and an adherent and proinflammatory nature, sharing common features and pathways with inflammation in CIMP-H CRC [64], [163]. Additionally, *Fusobacterium* might induce release of reactive oxygen species leading to chronic inflammation, possibly leading to high levels of aberrant base 7,8-dihydro-8-oxo-guanine (8-oxoG), most commonly modified by reactive oxygen species [164]. FN may also lead to development of CIMP-H CRC through possible activation of NF-κB, a transcription factor that is a key regulator of gene expression associated with tumor growth and an important link between inflammation and cancer [168]. Future studies should correlate diet history with levels of *Fusobacterium* in CIMP-H patients to explore the possible influence of diet on metabolites and microenvironment that influence development of CIMP tumors [169]. Additionally, utilizing evidence from metagenomic studies, utilizing gene markers of FN, namely, butyryl-CoA dehydrogenase, may prove a vital biomarker in predicting development of CIMP-H CRCs [170].

We also found CIMP-H tumors to be associated with advanced T-stage (T3/T4) among CRC patients. T staging is an important predictor of cancer outcomes, with advanced T staging indicating greater invasion of the tumor through colorectum and with higher T-stage indicating invasion through muscularis propria into pericolorectal tissues or visceral peritoneum. Though no clear evidence/basis exists underlying this mechanism, limited studies have identified CIMP-H tumors to have greater diameter/size as compared to CIMP-0 tumors [52], [54]. Finally, we found that CIMP-H was inversely associated with black race (compared with white). Blacks face a disproportionately higher burden of CRC as compared to other racial/ethnic groups in the United States [171]. Possible factors underlying these differences include genetic factors, lifestyle factors, cancer screening behaviors, and geographical variations as well as differences in gene expression underlying inflammatory pathways [171], [172]. Previous studies have shown differences in the incidence of CIMP-H between people of Anglo-Celtic origin and those of southern European origin [131]. Hence, further studies are needed to identify genetic basis for differences in prevalence of CIMP-H phenotype across racial groups. However, these analyses were based on data from three studies and hence need further validation.

Different molecular subtypes of CRC may have different environmental, genetic, and lifestyle risk factors, and investigation of possible risk factors for each molecular subtype may lead to a better understanding of how to prevent the disease [131]. Our findings that CIMP-H is associated with TILS, LVI, Crohn-like infiltrates, and *Fusobacterium nucleatum* highlight the role of the immune system mechanisms on development of CIMP-H. Many of these characteristics might be attributed to MSI-high status which is characterized by a strong immune response and possibly good prognosis in patients with high-MSI cancers. However, the association of CIMP status with clinical outcomes (overall survival) shows a trend towards poor survival, although it remains inconsistent owing to differences in methodologies, patient characteristics, and variables utilized in multivariable analysis [173]. Hence, the association of CIMP with these characteristics, including clinical outcomes, should be measured after stratifying by MSI status to understand the true effect of CIMP in CRC population [174].

Our study has several strengths and limitations. Our search strategy was broad and was not restricted to any specific methodology for measuring CIMP. The search was conducted using systematic Excel workbooks especially developed for systematic reviews. Training and discussions ensured that we incorporated and identified relevant articles. Finally, we excluded all articles on precancerous lesions. A major limitation in CIMP research is the lack of a standard definition for measuring and quantifying CIMP in CRC patients [8]. We did a comprehensive abstraction of data to allow for maximum understanding of CIMP in CRC. We found considerable heterogeneity in our measured associations, which can be attributed to differences in patient selection, racial or ethnic differences in CRC, tools or methods used for measuring CIMP, confounding by other factors such as lifestyle or family history, and differences in tissue preservation techniques [8]. In addition, CRC incidence varies across racial and ethnic groups and global regions, and we did not restrict our search to any region or country. For meta-analysis with less than 10 studies, results should be interpreted more cautiously as Egger test has low power to perform accurate bias assessments with <10 studies. Finally, no gold standard exists for assessing quality of nonobservational studies.

In summary, our systematic review provides a comprehensive assessment of CIMP in CRC and highlights distinct characteristics that define CIMP. While limited or small-scale studies had previously investigated these associations individually across individual studies, using the meta-analysis methods, we observed potential associations that warrant further exploration and validation in larger studies. Additionally, many of these characteristics are shared among high-MSI cancers, and future studies should assess these associations stratified by MSI status to study the molecular pathways impacting CIMP tumors independent of MSI status.

The following are the supplementary data related to this article.

Appendix 1 Search Strategies CRC-CIMP Appendix 1

Appendix 2 Forest Plots and Funnel Plots Appendix 2

Appendix 3 Cohort Studies Assessment Appendix 3

Appendix 4 Case Control Studies Assessment Appendix 4

## Additional Information

All manuscripts must contain an Additional Information section and should include the appropriate headings from the list below:

• Ethics approval and consent to participate: The study was approved by the University of Texas Health Science Center Institutional Review Board.
• Consent for publication: Not applicable.
• Availability of data and material: All necessary publications have been cited in this manuscript.
• Conflict of interest: No conflicts of interest to disclose.
• Funding: We would like to acknowledge following grant: NIH P30-CA016672 for supporting this project.
• Authors' contributions

1. Shailesh Advani: conceptualization, screening of articles, writing, data analysis
2. Pragati Advani: conceptualization, screening of articles, writing, data analysis
3. Stacia DeSantis: writing, data analysis
4. Derek Brown: writing, data analysis
5. Helena VonVille: conceptualization, writing, data analysis
6. Michael Lam: conceptualization, writing
7. Jonathan Loree: conceptualization, writing
8. Amir Mehrvarz Sarshekeh: conceptualization, writing
9. Jan Bressler: conceptualization, writing
10. David S. Lopez: conceptualization, writing
11. Carrie-Daniel MacDougall: conceptualization, writing
12. Michael D. Swartz: conceptualization, writing
13. Scott Kopetz: conceptualization, writing, data analysis
