# Supplementary material for: Clinical, Pathological, and Molecular Characteristics of CpG Island Methylator Phenotype in Colorectal Cancer: A Systematic Review and Meta-analysis
Source: Transl Oncol. 2018 Jul 30;11(5):1188–201. doi: 10.1016/j.tranon.2018.07.008 (PMC6080640; doi:10.1016/j.tranon.2018.07.008)
Supplement: Appendix 2 — Forest Plots and Funnel Plots [file mmc2.docx]

**Appendix 3: This document provides outputs for funnel and forest plots for association of CIMP with demographic, clinical, pathological and mutational/molecular characteristics**

1. **Age:**

Figure 1: Funnel Plot for studying the association of CIMP with age in CRC patients.

Figure 2: Forest Plot for studying the association of CIMP with age in CRC patients Race

1. Race

Figure 3: Funnel plot for assessing publication bias in the association of CIMP with African American race

Figure 4: Forest plot for studying the association of CIMP with African American race in CRC patients.

Figure 5: Funnel plot for assessing publication bias in the association of CIMP with Hispanic race

Figure 6: Forest plot for studying the association of CIMP with Hispanic race in CRC patients.

1. **Gender:**

Figure 7: Funnel plot for assessing publication bias in the association of CIMP with female gender

Figure 8: Forest plot for studying the association of CIMP with female gender in CRC patients.

1. **Family History of CRC (N=4 Studies)**

Figure 9: Funnel plot for assessing publication bias in the association of CIMP with family history of CRC

Figure 10: Forest plot for studying the association of CIMP with family history of CRC in CRC patients.

**Clinical Characteristics:**

1. **Right/Proximal vs Left/Distal+Rectum**

Figure 11: Funnel plot for assessing publication bias in the association of CIMP with localization.

Figure 12: Forest plot for studying the association of CIMP with localization in CRC patients.

1. **Staging System: OR (Stage 3/4 vs Stage 1/2)**

Figure 13: Funnel plot for assessing publication bias in the association of CIMP with overall staging

Figure 14: Forest plot for studying the association of CIMP with overall staging in CRC patients.

1. **T-Staging**

Figure 15: Funnel plot for assessing publication bias in the association of CIMP with T-staging.

Figure 16: Forest plot for studying the association of CIMP with T-staging (T3/T4 vs T1/T2) in CRC patients.

1. N Staging:

Figure 17: Funnel plot for assessing publication bias in the association of CIMP with N-staging.

Figure 18: Forest plot for studying the association of CIMP with N-staging (N1/N2 vs N0) in CRC patients.

1. **M Staging:**

Figure 19: Funnel plot for assessing publication bias in the association of CIMP with M-staging.

Figure 20: Forest plot for studying the association of CIMP with M-staging (M1/2 vs M0) in CRC patients.

1. **Synchronous CRC**

Figure 21: Funnel plot for assessing publication bias in the association of CIMP with Synchronous CRC

Figure 22: Forest plot for studying the association of CIMP with Synchronous CRC in CRC patients.

1. **Liver Mets**

Figure 23: Funnel plot for assessing publication bias in the association of CIMP with liver metastases

Figure 24: Forest plot for studying the association of CIMP with liver metastases in CRC patients.

**Pathological Characteristics:**

1. Lymphovascular Invasion:

Figure 25: Funnel plot for assessing publication bias in the association of CIMP with lymphovascular invasion.

Figure 26: Forest plot for studying the association of CIMP with lymphovascular invasion in CRC patients.

1. **Tumor Infiltrating Lymphocytes:**

Figure 27: Funnel plot for assessing publication bias in the association of CIMP with tumor infiltrating lymphocytes (TILS)

Figure 28: Forest plot for studying the association of CIMP with tumor infiltrating lymphocytes (TILS) in CRC patients.

1. **Peritumoral Lymphocytes**

Figure 29: Funnel plot for assessing publication bias in the association of CIMP with peritumoral lymphocytes

Figure 30: Forest plot for studying the association of CIMP with peritumoral lymphocytes in CRC patients.

1. **Vascular Invasion/Infiltration**

Figure 31: Funnel plot for assessing publication bias in the association of CIMP with vascular invasion

Figure 32: Forest plot for studying the association of CIMP with vascular invasion lymphocytes in CRC patients.

1. **Mucinous Characteristics:**

Figure 33: Funnel plot for assessing publication bias in the association of CIMP with mucinous histology

Figure 34: Forest plot for studying the association of CIMP with mucinous histology in CRC patients.

1. **Perineural Invasion**

Figure 35: Funnel plot for assessing publication bias in the association of CIMP with perineural invasion

Figure 36: Forest plot for studying the association of CIMP with perineural invasion in CRC patients.

1. **Signet Ring Cell features**

Figure 37: Funnel plot for assessing publication bias in the association of CIMP with signet ring cell features

Figure 38: Forest plot for studying the association of CIMP with signet ring cell features in CRC patients.

1. **Crohns Like Infitrate:**

Figure 39: Funnel plot for assessing publication bias in the association of CIMP with Crohn’s Like Infiltrate

Figure 40: Forest plot for studying the association of CIMP with Crohn’s Like Infiltrate race in CRC patients.

1. **Differentiation:**

Figure 41: Forest plot for studying the association of CIMP with differentiation in CRC patients.

Figure 42: Funnel plot for studying the association of CIMP with differentiation in CRC patients.

**Mutational and Molecular Characteristics:**

1. **TP53 Mutation:**

Figure 43: Funnel plot for assessing publication bias in the association of CIMP with Tp53 mutation

Figure 44: Forest plot for the association of CIMP with Tp53 Mutation

1. **KRAS Mutation**

Figure 45: Funnel plot for assessing publication bias in the association of CIMP with KRAS mutation

Figure 46: Forest plot for the association of CIMP with KRAS mutation in CRC patients

1. **BRAF Mutation:**

Figure 47: Funnel plot for assessing publication in the association of CIMP with BRAF mutation

Figure 48: Forest plot for studying association of CIMP with BRAF mutation

1. **PIK3CA**

Figure 49: Funnel plot for assessing publication bias in the association of CIMP with PIK3CA mutation

Figure 50: Forest plot for studying the association of CIMP with PIK3CA mutation in CRC patients.

1. APC Mutation:

Figure 51: Funnel plot for assessing publication bias in the association of CIMP with Tp53 mutation

Figure 52: Forest plots for the association of CIMP with APC mutation in CRC patients.

1. **Microsatellite Instability (MSI-H)**

Figure 53: Funnel plot to assess publication bias in the association of CIMP with MSI-High among CRC patients.

Figure 54: Forest Plot for CIMP-H with MSI-High.

1. **Fusobacterium Nucleatum:**

Figure 55: Funnel plot for assessing publication bias in the association of CIMP with Fusobacterium Nucleatum

Figure 56: Forest plot for studying the association of CIMP with Fusobacterium Nucleatum in CRC patients.
