# Supplementary material for: Clinical, Pathological, and Molecular Characteristics of CpG Island Methylator Phenotype in Colorectal Cancer: A Systematic Review and Meta-analysis
Source: Transl Oncol. 2018 Jul 30;11(5):1188–201. doi: 10.1016/j.tranon.2018.07.008 (PMC6080640; doi:10.1016/j.tranon.2018.07.008)
Supplement: Appendix 3 — Cohort Studies Assessment [file mmc3.docx]

| Quality Assessment of Cohort Studies Using the NewCastle-Ottawa | | | | | | |
| --- | --- | --- | --- | --- | --- | --- |
| Author (Year) | Selection | Selection | Comparability | Comparability | Outcome | Outcome |
| Ahn (2005) | 3 |  | 1 |  | 3 |  |
| Alvi (2017) | 2 |  | 0 |  | 3 |  |
| An (2010) | 3 |  | 1 |  | 3 |  |
| Anacleto (2005) | 2 |  | 0 |  | 2 |  |
| Ang (2010) | 4 |  | 1 |  | 3 |  |
| Arain (2010) | 4 |  | 2 |  | 3 |  |
| Bae (2011) | 2 |  | 1 |  | 2 |  |
| Bae (2013) | 4 |  | 1 |  | 2 |  |
| Bae (2016) | 3 |  | 1 |  | 3 |  |
| Barault (2008) | 3 |  | 1 |  | 3 |  |
| Beg (2015) | 3 |  | 1 |  | 3 |  |
| Bond (2012) | 4 |  | 2 |  | 3 |  |
| Bond (2012) | 4 |  | 2 |  | 3 |  |
| Bond (2015) | 2 |  | 1 |  | 2 |  |
| Bruin (2011) | 2 |  | 1 |  | 2 |  |
| Chen (2016) | 4 |  | 1 |  | 3 |  |
| Cleven (2014) | 4 |  | 1 |  | 2 |  |
| Dahlin (2008) | 4 |  | 1 |  | 3 |  |
| Day (2013) | 3 |  | 1 |  | 3 |  |
| de Vogel (2009) | 4 |  | 2 |  | 3 |  |
| Deng (2008) | 2 |  | 1 |  | 2 |  |
| Donada (2013) | 2 |  | 1 |  | 3 |  |
| Edin (2012) | 4 |  | 2 |  | 3 |  |
| English (2008) | 4 |  | 2 |  | 3 |  |
| Etienne-Grimaldi (2010) | 3 |  | 1 |  | 3 |  |
| Ferracin (2008) | 2 |  | 1 |  | 2 |  |
| Fu (2016) | 2 |  | 1 |  | 3 |  |
| Gonzalo (2014) | 2 |  | 0 |  | 1 |  |
| Hawkins (2002) | 3 |  | 2 |  | 3 |  |
| Hinoue (2012) | 3 |  | 2 |  | 2 |  |
| Hokazono (2014) | 3 |  | 2 |  | 3 |  |
| Hughes (2011) | 4 |  | 1 |  | 3 |  |
| Iacopetta (2007) | 3 |  | 1 |  | 3 |  |
| Ibrahim (2010) | 3 |  | 0 |  | 3 |  |
| Imamura (2014) | 4 |  | 2 |  | 3 |  |
| Inamura (2014) | 4 |  | 2 |  | 3 |  |
| Inamura (2015) | 4 |  | 2 |  | 3 |  |
| Ito (2015) | 2 |  | 1 |  | 2 |  |
| Jo (2012) | 2 |  | 1 |  | 3 |  |
| Jorrisen (2015) | 3 |  | 1 |  | 3 |  |
| Jover (2011) | 4 |  | 1 |  | 3 |  |
| Kakar (2008) | 2 |  | 1 |  | 3 |  |
| Kakar (2012) | 1 |  | 1 |  | 1 |  |
| Kang (2015) | 2 |  | 1 |  | 2 |  |
| Karpinski (2011) | 2 |  | 1 |  | 1 |  |
| Kawasaki (2008) | 4 |  | 2 |  | 3 |  |
| Kim (2005) | 1 |  | 0 |  | 0 |  |
| Kim (2010) | 3 |  | 1 |  | 2 |  |
| Kim (2017) | 3 |  | 2 |  | 3 |  |
| Kohonen-Corish (2014) | 3 |  | 1 |  | 3 |  |
| Kokellar (2017) | 3 |  | 1 |  | 2 |  |
| Konishi (2011) | 3 |  | 1 |  | 2 |  |
| Laskar (2015) | 3 |  | 1 |  | 2 |  |
| Lee (2008) | 4 |  | 1 |  | 3 |  |
| Li (2014) | 3 |  | 1 |  | 3 |  |
| Liao (2012) | 4 |  | 2 |  | 3 |  |
| Luo (2014) | 2 |  | 1 |  | 3 |  |
| Maeda (2011) | 2 |  | 1 |  | 1 |  |
| Marisa (2013) | 4 |  | 1 |  | 3 |  |
| McInnes (2017) | 3 |  | 1 |  | 2 |  |
| Messick (2010) | 1 |  | 1 |  | 2 |  |
| Mima (2015) | 4 |  | 2 |  | 3 |  |
| Min (2011) | 3 |  | 1 |  | 3 |  |
| Minarikova (2016) | 2 |  | 1 |  | 1 |  |
| Morikawa (2011) | 4 |  | 2 |  | 3 |  |
| Nosho (2008) | 1 |  | 0 |  | 1 |  |
| Nosho (2008) | 4 |  | 2 |  | 3 |  |
| Nosho (2009) | 4 |  | 2 |  | 3 |  |
| Ogino (2007) | 4 |  | 2 |  | 3 |  |
| Ostwald (2009) | 2 |  | 0 |  | 2 |  |
| Oyama (2004) | 2 |  | 1 |  | 2 |  |
| Park (2017) | 2 |  | 1 |  | 2 |  |
| Perea (2014) | 3 |  | 1 |  | 3 |  |
| Perea (2015) | 2 |  | 0 |  | 1 |  |
| Rosty (2013) | 4 |  | 2 |  | 3 |  |
| Saadallah-Kallel (2017) | 3 |  | 1 |  | 2 |  |
| Samadder (2013) | 3 |  | 1 |  | 3 |  |
| Samowitz (2005) | 4 |  | 2 |  | 3 |  |
| Samowitz (2007) | 3 |  | 1 |  | 3 |  |
| Sanchez (2009) | 3 |  | 2 |  | 3 |  |
| Sawada (2016) | 2 |  | 1 |  | 1 |  |
| Seth (2009) | 2 |  | 0 |  | 2 |  |
| Shen (2003) | 2 |  | 0 |  | 1 |  |
| Shiovitz (2014) | 3 |  | 1 |  | 3 |  |
| Silver (2011) | 3 |  | 1 |  | 3 |  |
| Simons (2013) | 4 |  | 2 |  | 3 |  |
| Siraj (2015) | 3 |  | 1 |  | 2 |  |
| Slattery (2006) | 4 |  | 2 |  | 3 |  |
| Slattery (2016) | 4 |  | 2 |  | 3 |  |
| Suehiro (2008) | 3 |  | 1 |  | 1 |  |
| Sugai (2006) | 2 |  | 1 |  | 1 |  |
| Tahara (2014) | 4 |  | 2 |  | 3 |  |
| Tanaka (2006) | 2 |  | 0 |  | 3 |  |
| Tanaka (2010) | 4 |  | 2 |  | 3 |  |
| TCGA (2012) | 4 |  | 2 |  | 3 |  |
| Tsai (2015) | 2 |  | 1 |  | 3 |  |
| van Rijnsoever (2002) | 2 |  | 0 |  | 2 |  |
| Van Roon (2010) | 2 |  | 0 |  | 2 |  |
| Vedeld (2017) | 4 |  | 2 |  | 3 |  |
| Walsh (2013) | 4 |  | 1 |  | 3 |  |
| Wang (2014) | 2 |  | 1 |  | 3 |  |
| Ward (2004) | 4 |  | 2 |  | 3 |  |
| Weisenberg (2006) | 2 |  | 2 |  | 3 |  |
| Whitehall (2012) | 4 |  | 2 |  | 3 |  |
| Williamson (2017) | 3 |  | 1 |  | 3 |  |
| Yagi (2010) | 3 |  | 1 |  | 2 |  |
| Yamamato (2002) | 1 |  | 1 |  | 1 |  |
| Yamamato (2012) | 3 |  | 1 |  | 1 |  |
| Yamashita (2003) | 3 |  | 1 |  | 3 |  |
| Yamuchi (2011) | 4 |  | 2 |  | 3 |  |
| Yang (2012) | 2 |  | 1 |  | 2 |  |
| Zhang (2016) | 3 |  | 1 |  | 3 |  |
| Zlobec (2011) | 4 |  | 2 |  | 3 |  |
