# Supplementary material for: Clinical, Pathological, and Molecular Characteristics of CpG Island Methylator Phenotype in Colorectal Cancer: A Systematic Review and Meta-analysis
Source: Transl Oncol. 2018 Jul 30;11(5):1188–201. doi: 10.1016/j.tranon.2018.07.008 (PMC6080640; doi:10.1016/j.tranon.2018.07.008)
Supplement: Appendix 4 — Case Control Studies Assessment [file mmc4.docx]

| Appendix 5: Case Control Study Quality Assessment Using the NewCastle-Ottawa Scale | | | | | | |
| --- | --- | --- | --- | --- | --- | --- |
| Author (Year) | Selection | Selection | Comparability | Comparability | Exposure | Exposure |
| Ang (2008) | 2 |  | 2 |  | 2 |  |
| Curtin (2007) | 4 |  | 2 |  | 3 |  |
| Jia (2016) | 2 |  | 1 |  | 2 |  |
| Karpinski (2012) | 2 |  | 1 |  | 2 |  |
| Saambudash (2017) | 4 |  | 2 |  | 2 |  |
| Samowitz (2006) | 4 |  | 2 |  | 3 |  |
| Van Guelpen (2010) | 4 |  | 2 |  | 3 |  |
| Weisenberg (2015) | 4 |  | 2 |  | 3 |  |
